# Language evolution and complexity considerations: The no half-Merge fallacy

**DOI:** 10.1371/journal.pbio.3000389

**Published:** 2019-11-27

**Authors:** Pedro Tiago Martins, Cedric Boeckx

**Affiliations:** 1 Section of General Linguistics, University of Barcelona, Barcelona, Spain; 2 University of Barcelona Institute for Complex Systems (UBICS), Barcelona, Spain; 3 Catalan Institute for Advanced Studies and Research (ICREA), Barcelona, Spain

## Abstract

Recently, prominent theoretical linguists have argued for an explicit scenario for the evolution of the human language capacity on the basis of its computational properties. Concretely, the simplicity of a minimalist formulation of the operation Merge, which allows humans to recursively compute hierarchical relations in language, has been used to promote a sudden-emergence, single-mutation scenario. In support of this view, Merge is said to be either fully present or fully absent: one cannot have half-Merge. On this basis, it is inferred that the emergence of our fully fledged language capacity had to be sudden. Thus, proponents of this view draw a parallelism between the formal complexity of the operation at the computational level and the number of evolutionary steps it must imply. Here, we examine this argument in detail and show that the jump from the atomicity of Merge to a single-mutation scenario is not valid and therefore cannot be used as justification for a theory of language evolution along those lines.

## Abstract 2

This Essay argues that the evolution of language cannot be as simple as prominent linguists Berwick and Chomsky have recently proposed, showing that the jump from the atomicity of Merge to a single-mutation scenario is not valid, and therefore cannot be used as justification for theory of language evolution along those lines.

## Introduction

The capacity for language is a defining trait of the human species. Understanding the nature of this capacity and how it came to be is a major topic of research (see [1] for a recent special issue on the topic). A leading proposal on the nature of the capacity, coming from the work of Chomsky [2], is that humans are equipped with some form of innate circuitry that allows for recursive computation over hierarchical structures. The theory describing this capacity has changed over the decades, with the most recent major articulation [3] proposing a basic operation named Merge. In its minimal expression, this operation takes two linguistic units (say, *α* and *β*) and forms a set {*α,β*}, which can, in turn, function as a unit to be further combined: {…{*γ*,{*α,β*}}…}. For example, Merge can take the units *the* and *book* and form the set {*the*, *book*} and further merge that set with *bought* and form the set {*bought*, {*the*, *book*}} and so on. Merge is claimed to be sufficient to yield grammatical structure and to be unique to humans.

As for the question of evolution, in a recent book, Berwick and Chomsky [4] propose that Merge, being such a simple operation, had to be the result of a single genetic mutation that endowed one individual with the necessary biological equipment for language. This idea is also defended in other recent work (e.g., [5–7]).

There are different parts to the position in [4], to which we will return briefly. But the key argument that interests us here is the claim that, because Merge is either fully present or fully absent, the human language faculty had to emerge suddenly, as the result of a single mutation.

The argument here is that because there can be no intermediate steps between "not having Merge" and "having Merge" as a formal operation underlying recursion—in other words, there cannot be such a thing as half-Merge—there can be no multiple, gradual evolutionary steps accounting for its emergence. Thus, Merge and, with it, a full-blown modern language faculty must have been the result of a sudden, single mutation. We will call this evolutionary scenario the "no half-Merge" argument.

In what follows, we will analyze this argument and show that it rests on tenets that do not hold (thus becoming the "no half*-*Merge fallacy"). We will conclude that this argument cannot be used as justification for a single-mutant theory of Merge, nor of human language, and that a different view is warranted.

## The no half-Merge argument

The single-mutant theory of language evolution in [4] rests on a number of points that are presented as tightly connected. In a nutshell: there was a Great Leap Forward, an unprecedented explosion of symbolic capacity and production sometime between the appearance of anatomically modern humans and a single exodus from Africa, roughly 100,000 years ago [4]. This can only be explained by a sudden (and single) genetic change that endowed one or a very small number of individuals with very advantageous capacities, the clearest expression of which are reflected in language. The actual result of that change was the operation Merge. This operation is said to be optimal and undecomposable. Furthermore, the authors in [4] state that things could not have happened otherwise, because there was not enough time for a more complex multistep evolutionary scenario to happen in a short time span. It is very important for this proposal for each of these tenets to hold, for one rests upon the other.

Even though the present paper focuses on the atomicity of Merge and its evolutionary implications, we see evidence for doubting the other strands of the evolutionary narrative in [4]. The Great Leap Forward, single-group exodus out-of-Africa narrative, taken for granted in [4], has lost its original appeal, with mounting evidence in favor of a multigroup, multistep evolutionary trajectory of *Homo sapiens* [8–10]. Recent work has put forward models that are more consistent with the diversity evident in the fossil record, advancing the idea that several populations from different regions within Africa gave rise to anatomically modern humans [10–12]. The out-of-Africa exodus, which, it is now thought, did not consist of a single event, has been pushed as far back as approximately 120,000 years ago, because fossils do not fit the original timeline [13]. The chronologically staggered and dispersed nature of the archaeological record used to infer cognitive modernity also points to this view [8, 14]. Moreover, many of the artifacts once associated with *H*. *sapiens*' cognitive modernity have been attributed to then-coexisting human species [15].

The idea that Merge was the result of a single mutation and that there was not enough time for multiple mutations to give rise to it has recently been modeled, and, contrary to expectations, a multistep scenario turns out to be much more plausible [16]. The model in [16] is based on the assumptions of [4] and other information consistent with them, such as the presupposition of a single-mutation event, maximum population size at that time, the extremely large fitness advantage the change would confer, and number of offspring that would be expected. By using standard population genetic approaches (diffusion models [17] and extreme value theory [18]), the authors show that a single macromutation scenario is much less likely than one whereby several mutations have smaller fitness advantages. Therefore, there seems to be no independent evolutionary-dynamics motivation for the single-mutation scenario that in [4] is called the “simplest assumption.” Thus, it seems that both evolutionary dynamics and the inadequacy of the Great Leap Forward idea are independent reasons for doubting key aspects of the single-mutant theory of the evolution of language.

Let us then turn to another aspect of the proposal of [4], namely, that because Merge is atomic, it could only have evolved as the result of a single mutation, for this "phenotype" does not allow for intermediate steps. In [4], it is put as follows:

"A plausible speculation is that some small rewiring of the brain provided the core element of the Basic Property: an optimal computational procedure, which yields an infinite array of hierarchically structured expressions, each interpreted systematically at the conceptual interface with other cognitive systems. . . .It is, in fact, not easy to conceive of a different possibility, since there can be no series of small steps that leads to infinite yield."

The argument has been stated most succinctly (and endorsed) by [19], who makes the same inference from formal complexity (or simplicity) to evolutionary steps: "There’s no such thing as half-recursion. It’s an all or nothing software trick" (p. 290); "it’s not totally implausible that such a faculty might have come about in a single mutation, which we should probably call a macro-mutation" (p. 382).

We now focus on the argument itself and articulate the reasons why it can't be used to justify a single-mutant theory of language evolution. We think it is worth examining this argument in detail because, in our experience, this is presented as "the last bastion of retreat" for linguists when a scenario like [4] is challenged.

## The no half-Merge fallacy

The language phenotype is defined in [4] as equivalent to Merge. Under this view, theories of language evolution are theories of the evolution of Merge, and everything else is deemed peripheral.

Theories of language competence (that is, what goes on in the "head" of a speaker) rest mainly on formalization. Under the assumption that the system we are interested in is a biological one, formalizing a linguistic mechanism is equivalent to describing it at the computational level in the sense of David Marr's influential "three levels of analysis" [20]. The computational level describes what is being done. The other two levels are the algorithmic (how something is being done, by which processes) and the implementational (the physical implementation in the brain, and all the way down to the genome). It is recognized in the literature that the formal simplicity of an operation deemed crucial to language cannot be conflated with simplicity at the biological level [21, 22]. And yet, this is precisely what accounts like [4] do: they extend the atomicity of Merge (computational description) down to the implementational level (single neural circuit rewiring; single mutation).

**Fig 1:**
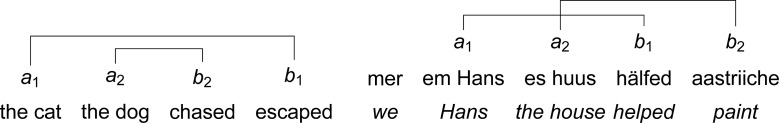
Nested dependencies (left) versus crossed dependencies (right). In the English example to the left, “*the cat the dog chased escaped*,” the dependencies do not cross. In the Swiss-German example (from [24]), to the right, “*mer Hans es huus hälfed aastriiche*” (we helped Hans paint the house), the dependencies cross.

An additional problem for an account like [4] concerns the simplicity of Merge (essentially, set formation, as described in the introduction). Such simplicity is only apparent: for Merge to adequately capture the core structural traits of linguistic competence, it must be formulated in such a way as to capture the distinction known to linguists as "external merge" (forming nested dependencies) and "internal Merge" (forming cross-serial/crossing dependencies) (Fig 1). Both kinds of dependencies occur in natural language, but the latter type, in which dependencies between items cross one another, requires more memory resources to keep track of all open dependencies across intervening elements [23].

If we go back to the hierarchy of formal languages [25] (Table 1), which we still take to be a useful categorization of the kinds of grammars that are computable, crossing dependencies were argued to require a level of complexity (mildly context sensitive) over and above that required for nested dependencies (context free). That is to say, crossing dependencies require more computational memory resources. Accordingly, they cannot simply be assumed to be part of the default Merge definition.

**Table 1:** The hierarchy of formal languages and corresponding automata.

Class	Grammar	Automaton
Type-3	Regular	Finite-state
Type-2	Context-free	Pushdown
Type-1	Context-sensitive	Linear bounded
Type-0	Unrestricted	Turing machine

Thus, it is perfectly reasonable to entertain a multistep scenario for Merge, with at least two steps: one step (effectively, external Merge in the terminology mentioned earlier) taking us beyond the range of resources attested in other species’ communication systems (limited to dependencies that can be captured by finite-state automata [26]). This would allow for the introduction of nested dependencies as described previously. A second step, corresponding to internal Merge, would make it possible for crossing dependencies to be part of the species’ communication system (technically corresponding to the characteristics of a linear-bounded automaton; Table 1).

Note, then, that even if we grant the claim that there is no such thing as half-recursion, it doesn't follow that Merge is equally atomic. It is perfectly possible that external-Merge and internal-Merge steps took place at different times, requiring at the very least two (macro)mutations. It is also possible under this view that only one of the macromutations would be unique to modern humans.

This is where Berwick and Chomsky's [4] argument concerning the evolution of Merge and the modern language faculty rests on the accuracy of the Great Leap Forward view and the claim that there was not enough evolutionary time to accumulate the relevant mutations. Even if we grant that there cannot be such a thing as half-external-Merge, the macromutation giving rise to it could have taken place thousands of years before other mutations could affect the brain in ways that gave rise to the computational regime supporting the internal-Merge step.

To be clear, we are not suggesting that it actually took exactly two steps for Merge to arise. We simply use Berwick and Chomsky's methodological approach to try to derive evolutionary steps by looking only at formal properties and conclude that these don't entail a single mutation. Besides the reasons we mention in the previous section, there seems to be no logical necessity for a scenario such as the one in [4].

The evolution of something as complex as human language deserves integration of results and insights from different corners of the research landscape, namely the fields of neurobiology, genetics, cognitive science, comparative biology, archaeology, psychology, and linguistics. This is hard because it requires compatible levels of granularity between all fields involved, but it is the only way of achieving meaningful understanding [27, 28]. This is where the a priori value of the single-mutant theory of language evolution of [4] lies. It offers a computational characterization of language that can serve as a boundary condition for other fields interested in addressing the evolutionary question. In the terms of [29], this potentially turns the question of language evolution into a "problem," as opposed to a "mystery."

Computational considerations must come to grips with both the insights and the data from other disciplines. In the case at hand, we believe theories of language evolution will benefit from taking on board the archaeological evidence questioning the Great Leap Forward. Similarly, the apparent simplicity of single-mutant scenarios should be reevaluated in light of simulations showing that multiple-mutation scenarios are more plausible, even over reduced temporal windows [16].

These empirical considerations are bypassed by work such as [4], and a single-mutation scenario is presented as arising from virtual necessity, because there cannot be such a thing as half-recursion. This eschews the fact that Merge is intended to capture a specific sense of recursion that encompasses the full range of dependencies in natural languages. Such dependencies have been known since the 1950s not to be uniform, something that the cover-term Merge obscures. This nonuniformity certainly admits a layered, mosaic-like evolutionary history.

**Fig 2:**
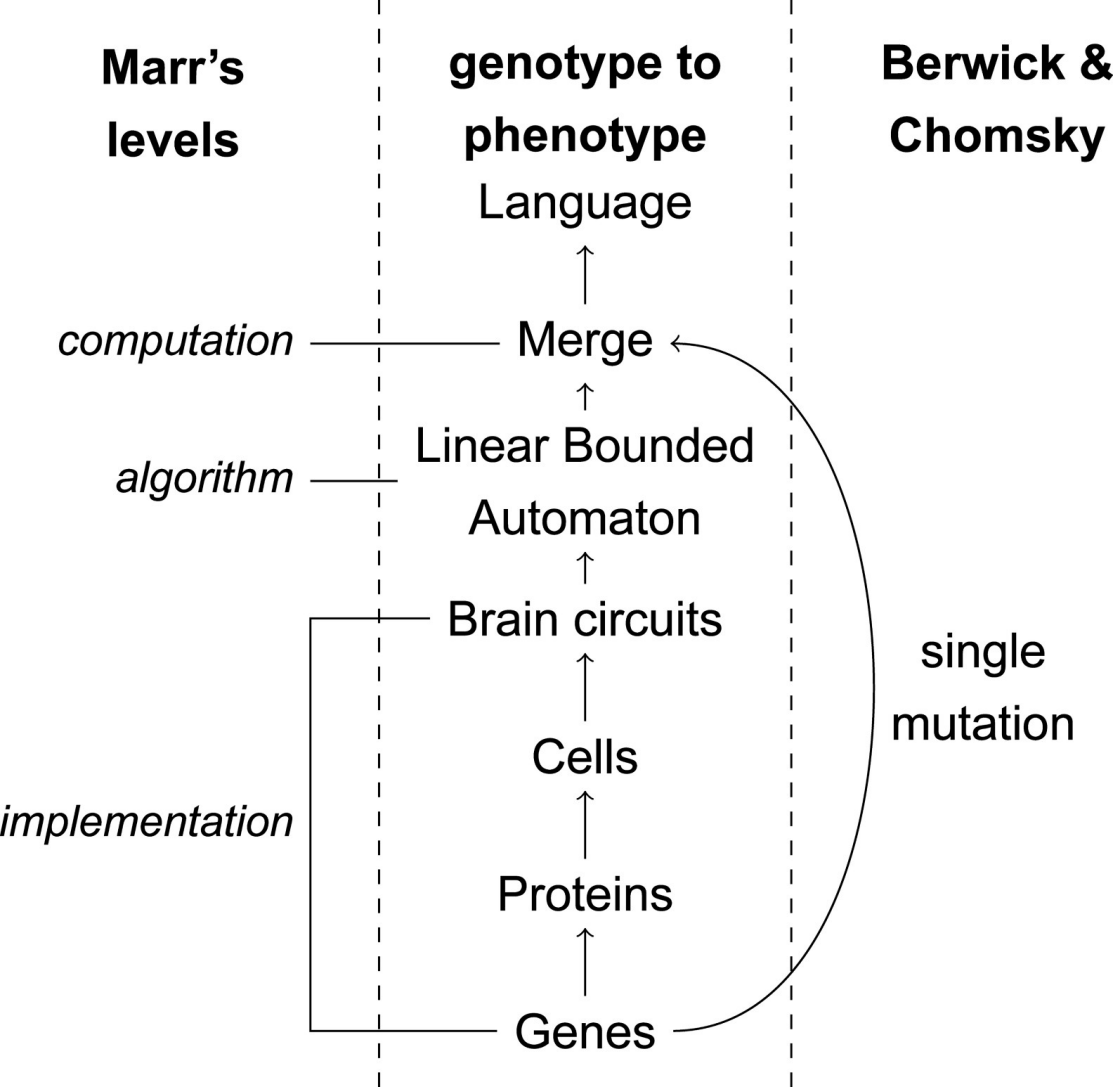
Berwick and Chomsky's theory of language evolution in the context of Marr's levels.

Marr's levels of analysis are of great importance to our understanding of cognitive traits. The implementational level, especially, must be given more attention than it has received in works such as [4] (Fig 2). This is the level at which the farthest-reaching claims are made when devising theories of the evolution of language. Eventually, the computational description must be linked to algorithmic and implementational descriptions that connect to the neural wetware and its molecular basis. Although this is an extremely hard problem, one can be fairly confident that there won't be a one-to-one mapping between the genotype and the phenotype [30]. Moreover, we now have the possibility to look at the complete catalog of changes between modern humans and archaic humans that reached fixation [31, 32], and it is unlikely that a single mental operation could be the direct result of any of these changes.

A single computational step need not correspond to a single-mutation or a single-rewiring event. In fact, to our knowledge, there is not a single case of a novel behavior arising from a single genetic mutation. Instead, each gene deemed important is but one cog in a network of genes [32, 33]. Even in domains that are easier to probe than cognition, such as concrete physical traits, it is extremely hard to find true evolutionary novelty and even more so to attribute it to single gene changes [34, 35].

We find it problematic to rely on "logical necessity" based on the formal complexity of a trait to motivate evolutionary scenarios. It is this fallacy that we draw attention to in this paper. If one were to follow the same logic, one would put forward single-mutation evolutionary scenarios for many phenotypic traits (say, counting or bipedalism), because it is hard to conceive of what the intermediate steps of the behavior might be (can there be such a thing as half-counting or half-bipedalism?). Evolutionary studies give us daily reasons to embrace complex scenarios, and we see no reason to abandon them in the context of language. Indeed, we think that decomposing the species-specific trait of modern language into a mosaic of less exceptional ingredients, each with its own evolutionary trajectory, is the only way to open inquiry into its emergence to empirical investigation.
